# Micafungin Is an Efficient Treatment of Multi Drug-Resistant *Candida glabrata* Urosepsis: A Case Report

**DOI:** 10.3390/jof7100800

**Published:** 2021-09-24

**Authors:** Zuzana Javorova Rihova, Lubica Slobodova, Anna Hrabovska

**Affiliations:** 1 Department of Clinical Pharmacology, Teaching Hospital Trnava, A. Zarnova 11, 917 75 Trnava, Slovakia; zuzana.javorova@fntt.sk; 2 Department of Pharmacology, Faculty of Medicine, Slovak Medical University in Bratislava, Limbova 12, 833 03 Bratislava, Slovakia; lubica.slobodova@szu.sk; 3 Department of Pharmacology and Toxicology, Faculty of Pharmacy, Comenius University in Bratislava, Odbojarov 10, 832 32 Bratislava, Slovakia; 4 Biomedical Research Centre, University Hospital Hradec Kralove, Sokolska 581, 500 05 Hradec Kralove, Czech Republic

**Keywords:** urosepsis, candiduria, candidemia, *Candida glabrata*, micafungin, echinocandins, multi drug-resistance

## Abstract

Candiduria is a common nosocomial infection in hospitalized patients, which may progress into life-threatening candidemia. Successful treatment of urosepsis requires early and effective antifungal therapy, while the available agents within three pharmacological classes each have characteristic pharmacokinetics and side effect profiles. Moreover, treatment of *Candida* spp. infections is becoming challenging due to increasing multi drug-resistance. Here, we present a case of candidemia resulting from a multi drug-resistant *C. glabrata* infection of the urinary tract. Due to resistance to fluconazole and a contraindication for amphotericin B, micafungin was used in the treatment, regardless of its unfavorable pharmacokinetic properties. Our study showed that despite the expected low levels in the urinary tract, micafungin was successful in the eradication of *C. glabrata* allowing full recovery of the patient. Thus, micafungin should be considered in the management of urosepsis caused by sensitive *Candida* spp.

## 1. Background

Candiduria is a common nosocomial infection that may progress into candidemia in susceptible patients, especially polymorbid, intensive care unit (ICU) patients [1,2,3]. The main risk factors of candiduria include exposure to invasive procedures (such as prolonged catheterization or urinary tract instrumentation), use of broad-spectrum antibiotics, extremes of age, female gender, diabetes mellitus, immunocompromised state, prolonged hospitalization, ICU stay, previous surgery, and abnormalities of the urinary tract [2,4,5]. Employing early, effective antifungal therapy is crucial for a favorable outcome of fungal urosepsis [6]. Three major classes of antifungal agents are available to clinicians, including azoles, echinocandins, and polyenes [7,8], with each having characteristic mechanisms of action, resistance patterns, pharmacokinetic (PK)/pharmacodynamics (PD) properties, and side effect profiles.

Azole antifungal agents have fungistatic properties, affecting the growth and proliferation of fungi cells by inhibiting ergosterol synthesis [8]. Unlike fluconazole, the other azole agents, such as itraconazole, posaconazole, and voriconazole, are metabolized in the liver and urinary excretion is either minimal or absent [9]. Therefore, these latter azole agents are not recommended for the treatment of *Candida* spp. urinary tract infections. Moreover, the increasing resistance of *Candida* spp. to azoles diminishes their utility in the treatment of candidiasis, including infections of the urinary tract [10].

Echinocandins are semisynthetic lipopeptides that inhibit the synthesis of an essential component of the fungal cell wall, 1,3-β-d-glucan, hence rendering them fungicidal [11]. The three available echinocandins, anidulafungin, caspofungin, and micafungin, have a broad spectrum of activity, including *Candida* species resistant to azoles and polyenes [10]. With the exception for *C. glabrata*, resistance to echinocandins remains low [10]. The use of echinocandins in uroinfections is however limited due to their PK characteristics, especially the high degree of protein binding and minimal distribution in urine [2,11]. 

Polyenes are large compounds with a macrolide structure that are derived from *Streptomyces* bacteria. With a broad spectrum of activity, these agents disrupt the structure of the fungal membrane by binding to ergosterol, thus causing cell death [8]. Amphotericin B is the only polyene antifungal drug indicated for systemic administration; however, its clinical use is limited by its nephrotoxic, hepatotoxic, and significant infusion-related adverse effects [8]. 

Despite numerous clinical studies [7,12], the clear superiority of one therapeutic agent over the other has not been proven. Here, we present a successful treatment of a polymorbid, ICU patient with candidemia resulting from a multi drug-resistant *C. glabrata* infection of the urinary tract. Due to resistance to fluconazole and a contraindication for amphotericin B, micafungin was used in the treatment, regardless of its unfavorable PK properties. Despite the expected low levels in the urinary tract [2], micafungin was successful in the eradication of *C. glabrata* in this case.

## 2. Case Report and Results

**Figure 1:**
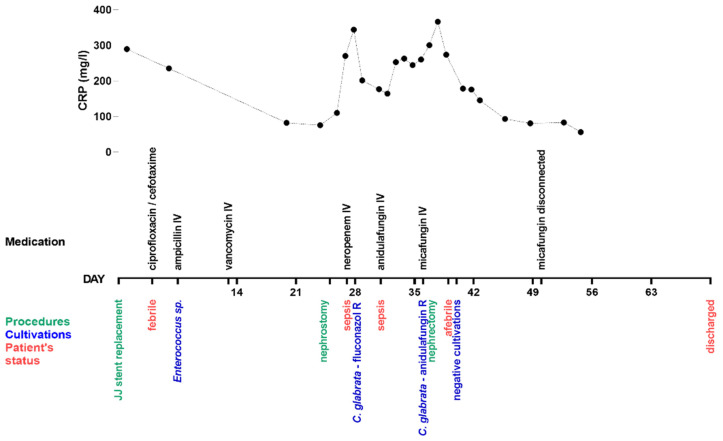
The timeline of case development. The timeline summarizes urinary tract instrumentation and surgical procedures (green), results from blood and urine cultivations (blue), patient’s status (red), medication (black) and a line plot of C-reactive protein (CRP) values (reference range 0–5 mg/L).

A 65-year-old female patient with metabolic syndrome, arterial hypertension, type 2 diabetes mellitus, visceral obesity, atrial fibrillation, chronic kidney disease (stage G4A2 KDIGO) with chronic tubulointerstitial nephritis, was admitted to urology for relapse of obstructive pyelonephritis and progression of chronic kidney disease four days after the ureteric stent (double J stent, JJ stent) replacement (day 0, d0). The patient was well known to the urology medical team due to frequent past hospitalizations related to stent complications, obstructive pyelonephritis, and recurrence of infections. Her post-JJ stent replacement recovery was protracted by febrile episodes, urinary retention, and rising CRP and leukocytosis. Empiric antimicrobial therapy with ciprofloxacin and cefotaxime was started. When urine culture/sensitivity data for *Enterococcus* sp. became available, the antimicrobial therapy was changed to ampicillin intravenously (IV) and later to vancomycin IV, which improved the patient’s clinical picture. On d25, the patient underwent JJ stent extraction and percutaneous nephrostomy. On d27, her rapidly deteriorating condition warranted an ICU transfer with probable urosepsis. Empiric broad spectrum antibacterial therapy with meropenem (500 mg IV) was started due to the likelihood of a nosocomial pathogen. A CT scan verified the presence of bilateral pyelonephritis with a possible abscess on the left kidney and suspected cystitis. The presence of an abscess was excluded by MRI. Her condition gradually improved, but on d31 she suffered a relapse of sepsis. Urine cultures obtained on d27 were positive for *C. glabrata* (identified by YST8, Ref. 1005, DIAGNOSTICS Slovakia; BBL™ CHROMagar™, Ref. PA-257480.05, Becton Dickinson Gmb, Germany), resistant to fluconazole, and the patient was empirically treated with echinocandin anidulafungin IV dosed according to the manufacturer’s recommendations per the summary of product characteristics (loading dose of 200 mg, thereafter 100 mg daily). Subsequently, the urine and blood cultures that were collected prior to the first dose of anidulafungin, confirmed *C. glabrata* in urine and in blood, sensitive to micafungin (MIC 0.015 mg/L) and amphotericin B (MIC 0.500 mg/L) and resistant to anidulafungin (MIC 0.125 mg/L), caspofungin (MIC 0.125 mg/L) and fluconazole (MIC 64 mg/L) (MICRONAUT-AM, Ref. E1-831-040, MERLIN Diagnostika GmbH, Germany). The isolate could not be classified as either sensible or resistant to voriconazole (MIC 0.500 mg/L), according to the EUCAST breakpoint tables for the interpretation of MICs. Antifungal therapy was changed to micafungin IV (100 mg daily) on d36 and based on the finding of *Staphylococcus hominis* in the same blood culture, antibacterial therapy continued with linezolid IV (1200 mg daily). On d36, the patient underwent left-sided nephrectomy. From d39, the patient remained afebrile and subjectively felt better. Blood cultures were repeated on d40 and all results, including the fungal culture, were negative. The patient received micafungin for 15 days after the last positive fungal blood culture, with the last dose of micafungin given on d50. The antibacterial drug therapy was changed repeatedly, according to bacterial findings in the urine cultures. Finally, the nephrectomy wound was healing, all repeated cultures were negative, and after a 70-day hospitalization, the patient was discharged. For a detailed timeline of the clinical status and medical interventions see Figure 1 and for the measured biochemical parameters see Table 1.

## 3. Discussion

Candidemia and invasive candidiasis affecting body fluids, deep tissues, and organs are serious ICU-acquired nosocomial infections. They are characterized by high morbidity, prolonged hospital stays with an increased intensive-care setting, and high mortality [13]. *C. albicans* is a predominant species in nosocomial and community-onset fungal infections worldwide, even though a steady decrease in frequency over the past 20 years has been reported [10]. The most common non-albicans species is *C. glabrata*. The SENTRY Antifungal Surveillance Program confirmed *C. glabrata* in 18.7% of all 20,788 *Candida* spp. isolates collected in North America, Europe, Latin America, and the Asia–Pacific region, while over a 20-year surveillance period, a steady increase (from 16% to 19.6%) was reported [10].

Here we present a patient with multiple risk factors, including urinary tract instrumentation, female gender, elderly age, diabetes mellitus, and prolonged stay at the ICU, who developed invasive candidiasis caused by *C. glabrata*.

The selection of an antifungal agent for the *Candida* spp. uroinfection treatment depends on the patient’s clinical status and co-morbidities, distribution in the urinary tract, local epidemiology data, character of the treatment (prophylactic, preemptive, empiric, or definitive), and, more importantly, the sensitivity/resistance of the species towards antifungal agents [5,14]. While resistance of *C. glabrata* to antifungal drugs is considered low, an increase in resistance to antifungal drugs has been observed [10]. Candida resistant to ≥1 agent in ≥2 drug classes is considered to be multi drug-resistant [15]. Multi drug-resistant *C. glabrata* has become common in many hospitals and medical centers and possesses serious management challenge [15,16]. Also in our case, cultivation results confirmed *C. glabrata* isolate to be multi drug-resistant (to the azole, fluconazole, and to the echinocandins, anidulafungin and caspofungin).

Resistance to fluconazole ruled out its use in the therapy of urosepsis in the presented case. Based on polymorbidity of the patient and poor clinical status, amphotericin B, the drug of choice for fluconazol-resistant candiduria [2], was contraindicated. Besides low urinal excretion of other azoles, voriconazole (IV) was also contraindicated in this patient with impaired renal functions due to the accumulation of vehicle (cyclodextrin). This left us with the last clinically available antifungal drug group-echinocandins. Nevertheless, the further confirmation of resistance to anidulafungin and caspofungin narrowed the choice to micafungin. This resistance characteristic is in line with the results from the SENTRY Antifungal Surveillance Program, which showed lower frequency of *C. glabrata* resistance to micafungin than the other two echinocandins [10]. There is a novel antifungal drug, Ibrexafungerp, which could have been an effective treatment for our patient. Nevertheless, it has only recently been approved by the U.S. Food and Drug Administration to treat vulvovaginal candidiasis and was unavailable during the time period of this case.

Micafungin is indicated for the treatment of candidemia, esophageal candidiasis, and prophylaxis of *Candida* spp. infections in immunocompromised patients [17]. It has very effective fungicidal activity against most *Candida* spp. Generally, the drug is well tolerated and its safety profile from the perspective of comorbidity or drug interactions is benign [17]. The drug has a poor distribution into the urinary bladder because it is largely metabolized by the liver, with some metabolites being pharmacologically active [9,17]. Less than 1% of the unchanged parent compound is excreted in urine. The drug penetrates quickly and well into renal tissue with a concentration approaching that of plasma levels. No dosage adjustment is required in patients with renal dysfunction [17]. As discussed above, micafungin was used based on cultivation results and the clinical status of the patient, but also due to evidence of a valid PK/PD argument supporting its effectiveness in cystitis and the antifungal drug availability at our institution. Our decision was supported by published case reports of successful treatment of patients with candiduria with micafungin IV [18,19].

Grau et al. [20] performed PK and PD experiments on six patients with urinary tract infections positive for *Candida* spp. All patients were successfully treated with micafungin IV. Eighty-three per cent of treated patients had urine micafungin PK:PD ratio of at least 4, sufficing for efficacy. The authors concluded that diligent monitoring of drug levels can guide micafungin dosing for optimal PK/PD parameters, leading to successful eradication of *Candida* pathogens [20]. Gabardi et al. [21] evaluated 33 hospitalized patients with candiduria receiving micafungin IV. In the majority of these cases, micafungin completely eradicated *Candida* spp. from the urine. The authors concluded that the effectiveness of micafungin in the eradication of *Candida* spp. may be due to a combination of rapid penetration into renal and bladder tissue together with its novel fungicidal mode of action [21].

## 4. Conclusions

We were successful in treating our patient with micafungin for *C. glabrata* urosepsis. Due to lack of availability, we did not monitor the therapeutic drug levels of micafungin and we did not perform any specific PK measurements. The patient tolerated the therapy, except for a transient skin rash of mild severity, and began to subjectively feel better relatively quickly. We suspect that personal hygiene incorrectly performed by the patient due to obesity was a likely contributing factor to her prolonged hospitalization with several setbacks and repeated positive urine cultures for nosocomial Gram negative bacteria strains. Our patient was discharged from the hospital asymptomatic and was doing well in ambulatory care.

## Figures and Tables

**Table 1:** Results from the biochemical analyses performed during the case development.

DAY.	1	6	24	27	32	35	39	41	46	49	53	62
leu ×10^−9^/L)	22.48	8.94	6.89	11.91	25.37	23.34	11.18	8.46	5.51	4.48	6.64	7.59
sCr (μmol/L)	390.2	513.9	287.2	488.6	397.7	324.3	344.7	289.1	266.9	273.4	277.2	278.7
bilirubin (μmol/L)			3.17					8.26	5.02	3.56		
AST (μkat/L)	0.95		0.57				0.3	0.45	0.64	0.53		
ALT (μkat/L)	0.8		0.75				0.14	0.19	0.36	0.41		
GMT (μkat/L)			1.07				1.58	1.38	0.96	0.58		
ALP (μkat/L)			2.98				4.23	3.53	2.74	2.64		

Reference ranges: leu-leukocytes (3.6–11 × 10^−9^/L), sCr- serum creatinine (53–97 μmol/L), bilirubin (5–21 μmol/L), AST—aspartate aminotransferase (0–0.52 μkat/L), ALT-alanine aminotransferase (0–0.52 μkat/L), GMT-glutamyltransferase (0–0.63 μkat/L), and ALP-alkaline phosphatase (0.75–2.15 μkat/L).

## Data Availability

All data generated or analyzed during this study are included in this published article.
